# Environmental pH and the Requirement for the Extrinsic Proteins of Photosystem II in the Function of Cyanobacterial Photosynthesis

**DOI:** 10.3389/fpls.2016.01135

**Published:** 2016-08-09

**Authors:** Jaz N. Morris, Julian J. Eaton-Rye, Tina C. Summerfield

**Affiliations:** ^1^Department of Botany, University of OtagoDunedin, New Zealand; ^2^Department of Biochemistry, University of OtagoDunedin, New Zealand

**Keywords:** assembly, extrinsic proteins, oxygen-evolving complex, pH, photosystem II, reactive oxygen species, thylakoid lumen

## Abstract

In one of the final stages of cyanobacterial Photosystem II (PS II) assembly, binding of up to four extrinsic proteins to PS II stabilizes the oxygen-evolving complex (OEC). Growth of cyanobacterial mutants deficient in certain combinations of these thylakoid-lumen-associated polypeptides is sensitive to changes in environmental pH, despite the physical separation of the membrane-embedded PS II complex from the external environment. In this perspective we discuss the effect of environmental pH on OEC function and photoautotrophic growth in cyanobacteria with reference to pH-sensitive PS II mutants lacking extrinsic proteins. We consider the possibilities that, compared to pH 10.0, pH 7.5 increases susceptibility to PS II-generated reactive oxygen species (ROS) causing photoinhibition and reducing PS II assembly in some mutants, and that perturbations to channels in the lumenal regions of PS II might alter the accessibility of water to the active site as well as egress of oxygen and protons to the thylakoid lumen. Reduced levels of PS II in these mutants, and reduced OEC activity arising from the disruption of substrate/product channels, could reduce the *trans*-thylakoid pH gradient (ΔpH), leading to the impairment of photosynthesis. Growth of some PS II mutants at pH 7.5 can be rescued by elevating CO_2_ levels, suggesting that the pH-sensitive phenotype might primarily be an indirect result of back-pressure in the electron transport chain that results in heightened production of ROS by the impaired photosystem.

## Introduction

Photosystem II (PS II) is a thylakoid membrane-bound protein complex that functions as a water-plastoquinone oxidoreductase in oxygenic phototrophs (Vinyard et al., 2013). In cyanobacteria, the mature PS II monomer contains at least 17 membrane-spanning subunits, of which seven are essential for PS II function, as well as up to four extrinsic, thylakoid-lumen-associated subunits (PsbO, PsbU, PsbV, and possibly CyanoQ), which are necessary for maximal rates of oxygen evolution (Shen, 2015; Heinz et al., 2016; Roose et al., 2016). The PS II extrinsic proteins, along with the lumenal domains of the intrinsic reaction center proteins D1 and D2 and the adjacent chlorophyll-binding core antenna proteins CP43 and CP47, form a protective environment around the site of the Mn_4_CaO_5_ cluster or oxygen-evolving complex (OEC) that catalyzes the water-splitting reaction (Shen, 2015).

The extrinsic proteins bind to the PS II monomer subsequent to assembly and photoactivation of the Mn_4_CaO_5_ cluster (Dasgupta et al., 2008; Nickelsen and Rengstl, 2013). Based on their locations in the X-ray-derived structure of PS II from *Thermosynechococcus vulcanus* (Umena et al., 2011; Suga et al., 2015), and extensive biochemical studies (reviewed in Bricker et al., 2012; Ifuku, 2015; Ifuku and Noguchi, 2016; Roose et al., 2016), it seems likely that PsbO and PsbV bind first: PsbO binds via interactions with loop E of CP47, loop E of CP43 and the C-terminus of both D1 and D2; PsbV binds via loop E of CP43 and the C-terminus of both D1 and D2. Subsequently, PsbU binds via PsbO, PsbV, loop E of CP47, loop E of CP43 as well as the C-terminus of both D1 and D2; finally, CyanoQ is predicted to bind via associations with PsbO and loop E of CP47. Although none of the extrinsic proteins provide direct ligands to the Mn_4_CaO_5_ cluster, they protect this site from the reductive environment of the lumen, and increase the affinity for the Ca^2+^ and Cl^-^ co-factors (reviewed in Bricker et al., 2012).

During light-driven photosynthetic electron transport, electrons are extracted in a series of oxidative ‘S’ state transitions (S_0_–S_4_) of the Mn_4_CaO_5_ cluster, resulting in the oxidation of two waters; in this process four electrons are transferred sequentially to the PS II reaction center P_680_ via Y_Z_ (D1:Tyr161), and one dioxygen molecule and four protons are released to the thylakoid lumen (Shen, 2015; Najafpour et al., 2016). The X-ray-derived structures of PS II from *T. vulcanus* and *T. elongatus* have revealed that extensive hydrophilic regions and hydrogen bond networks in both extrinsic and intrinsic proteins in the vicinity of the OEC may allow water transport to, and proton and molecular oxygen transport from, the catalytic center (Linke and Ho, 2014; Lorch et al., 2015; Vogt et al., 2015).

The buildup of protons in the lumen from PS II water-splitting contributes to the pH gradient (ΔpH) and membrane potential (Δψ) across the thylakoid membrane, which creates a proton electrochemical potential that is used to drive the ATP synthase catalyzed production of ATP. Additionally, protons are pumped into the lumen independently of PS II via NADPH dehydrogenase complexes involved in cyclic electron flow (CEF) around Photosystem I (PS I), respiration, and carbon uptake (Battchikova et al., 2011), and via plastoquinol oxidation by the cytochrome *b*_6_*f* complex (Kallas, 2012). As a consequence, the cyanobacterial thylakoid lumen pH is acidified in the light, by around two pH units, relative to the cytosolic pH (Belkin et al., 1987; Belkin and Packer, 1988). Although the pH microenvironment in the vicinity of PS II would be expected to be independent of environmental pH, changes in environmental pH do affect PS II. A number of mutants in the model strain *Synechocystis* sp. PCC 6803 (hereafter *Synechocystis* 6803), which are deficient in extrinsic proteins that stabilize the OEC, are obligate photoheterotrophs or photomixotrophs in pH 7.5-buffered growth media, but were observed to grow photoautotrophically at pH 10.0 (Eaton-Rye et al., 2003).

Despite ongoing interest in the transcriptomic and proteomic response to pH in cyanobacteria (Ohta et al., 2005; Kurian et al., 2006; Summerfield and Sherman, 2008; Zhang et al., 2009; Li et al., 2014; Matsuhashi et al., 2015), relatively few studies have investigated the role of environmental pH on the assembly of PS II, or on the photochemical and redox processes of the photosystem. Here, we offer a perspective regarding the effects of environmental pH on the function of PS II in cyanobacterial cells and propose a mechanism by which some mutations in the lumenal regions of PS II prevent photoautotrophic growth at pH 7.5.

## Growth of pH-Sensitive PS II Mutants

### Environmental pH Affects PS II

**FIGURE 1:**
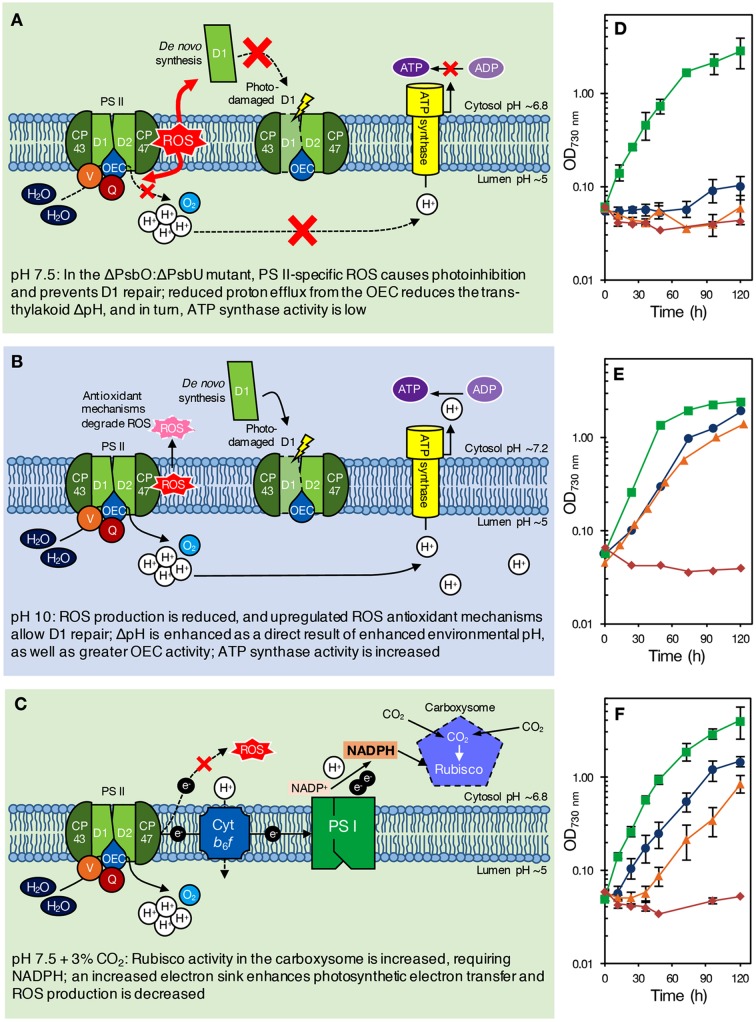
**(A–C)** Proposed model of pH effects on a ΔPsbO:ΔPsbU strain of *Synechocystis* 6803 at pH 7.5 **(A)**, green background; pH 10.0 **(B)**, blue background; and pH 7.5 with 3% CO_2_ **(C)**, green background. The ΔPsbO:ΔPsbU strain was chosen for illustrative purposes only; we would expect a similar response from other pH 7.5-sensitive strains, such as ΔPsbV:ΔCyanoQ. **(A)** At pH 7.5, ROS formation from an impaired OEC prevents PS II repair, causing photoinhibition and reducing PS II levels. Additionally, reduced delivery of protons to the thylakoid lumen results in low ΔpH and insufficient ATP synthase activity [alternatively, growth may be retarded via sensing of the reduced ΔpH (not shown)]. **(B)** At pH 10.0, however, upregulation of oxidative stress response genes induces the synthesis of antioxidant defense compounds, allowing PS II repair and photoautotrophic growth. Increased environmental pH naturally enhances ΔpH, increasing ATP synthase activity [or activating other ΔpH-dependent processes that promote photoautotrophic growth (not shown)]. **(C)** With 3% CO_2_, enhanced Rubisco activity in the carboxysome requires NADPH, drawing electrons from PS II that would otherwise lead to ROS production and photoinhibition. The internal pH values indicated are based on those determined for wild-type cells by Belkin et al. (1987) and Jiang et al. (2013), and might be different in PS II mutants. **(D–F)** Photoautotrophic growth of *Synechocystis* 6803 wild type, ΔPsbO:ΔPsbU, ΔPsbV:ΔCyanoQ, and ΔPsbO:ΔPsbV PS II mutants at **(D)** pH 7.5, ambient (∼0.04%) CO_2_ conditions; **(E)** pH 10.0, ambient (∼0.04%) CO_2_ conditions; and **(F)** pH 7.5, high (3%) CO_2_ conditions. Green, squares: wild type; blue, circles: ΔPsbO:ΔPsbU cells; orange, triangles: ΔPsbV:ΔCyanoQ cells; red, diamonds: ΔPsbO:ΔPsbV cells. Data in **(D,F)** are the mean of 3–7 independent experiments (±SEM, error bars not visible are smaller than the markers) and were carried out as described previously (Morris et al., 2014). Data in **(E)** (averages only) are derived from Eaton-Rye et al. (2003) and Summerfield et al. (2005a).

Many cyanobacterial species are able to grow photoautotrophically across a neutral to alkaline pH range, and oxygen evolution and PS II-specific variable chlorophyll fluorescence emission from *Synechocystis* 6803 wild-type cells was similar from pH 7.5–10.0 (Summerfield et al., 2013; Touloupakis et al., 2016). Across this pH range, the internal pH of cyanobacterial cells is well buffered by pH homeostasis mechanisms (Krulwich et al., 2011). For example, a relatively large change in external pH from pH 10.0 to 8.0 decreased cytosolic pH from 7.2 to 6.8 in *Synechocystis* 6803 (Jiang et al., 2013). In cyanobacteria, excluding thylakoid-deficient *Gloeobacter* spp., PS II extrinsic proteins and the oxygen-evolving machinery face the more acidic thylakoid lumen (pH = ∼5, Belkin et al., 1987) and are thus further protected from the environmental pH compared to the cytosol. Considering that the cytosol and thylakoid lumen are well-buffered with respect to environmental pH, and the fact that PS II oxygen evolution is known to function optimally when the lumen pH is relatively low, between pH 5.0 and 6.5 (Kramer et al., 1999; Najafpour et al., 2016), it was surprising that a number of *Synechocystis* 6803 PS II mutants were unable to grow photoautotrophically at environmental pH 7.5, whereas growth was possible at pH 10.0 (**Table 1**; **Figures 1D,E**) (Eaton-Rye et al., 2003; Summerfield et al., 2005a,b, 2007).

**Table 1:** Photoautotrophic growth, and relative level of PS II assembly of strains of *Synechocystis* sp. PCC 6803 carrying mutations in PS II extrinsic proteins, and lumenal domains of intrinsic proteins^1^.

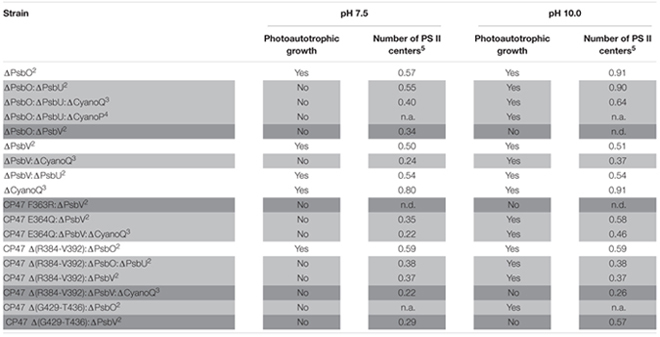

*^1^White rows: strains capable of photoautotrophic growth at pH 7.5 and pH 10.0; partially shaded rows: strains displaying the pH 7.5 sensitive phenotype; shaded rows: strains incapable of photoautotrophic growth at either pH. ^2^Eaton-Rye et al., 2003. ^3^Summerfield et al., 2005a. ^4^Summerfield et al., 2005b; n.a., data not available; n.d., no PS II could be detected. ^5^Number of PS II centers normalized to relative wild-type values in the same study and was determined using [^*14*^C] atrazine binding (Morgan et al., 1998).*

### PS II Mutants Lacking Extrinsic Proteins

The loss of extrinsic proteins has multiple effects on PS II (Bricker et al., 2012). By destabilizing the binding of Ca^2+^ and Cl^-^ the function of the Mn_4_CaO_5_ catalytic site might be directly affected (Ifuku and Noguchi, 2016). In addition, the extrinsic proteins, along with the lumenal domains of the intrinsic proteins, maintain channels that allow the access of substrate water to the catalytic site, and egress of oxygen and protons. The absence of either of the extrinsic PS II proteins PsbO and PsbV reduced growth and oxygen evolution compared to the *Synechocystis* 6803 wild type (Burnap and Sherman, 1991; Shen et al., 1995) but did not affect pH tolerance (Eaton-Rye et al., 2003). Deletion of PsbO or PsbV resulted in decreased oxygen evolution compared to deletion of PsbU or CyanoQ, consistent with the partial dependency of PsbU and CyanoQ binding on the presence of PsbO or PsbV (Shen et al., 1998; Thornton et al., 2004). Deletion of PsbU or CyanoQ in ΔPsbO or ΔPsbV backgrounds, respectively, revealed the pH-sensitive phenotype (**Table 1**, **Figures 1D,E**); ΔPsbO:ΔPsbU and ΔPsbV:ΔCyanoQ strains do not grow photoautotrophically at pH 7.5, whereas photoautotrophic growth at pH 10.0 is possible (Eaton-Rye et al., 2003; Summerfield et al., 2005a). Another extrinsic PS II protein, CyanoP, was also putatively assigned a role in the PS II dimer (Thornton et al., 2004; Nickelsen and Rengstl, 2013) but evidence suggests it may be a PS II assembly factor rather than a stoichiometric OEC subunit (Cormann et al., 2014; Jackson and Eaton-Rye, 2015). Deletion of CyanoP from other extrinsic protein mutants did not affect pH sensitivity (Summerfield et al., 2005b).

### PS II Mutants with Deletions and Amino Acid Substitutions in Intrinsic Proteins

Mutations in the large, lumenal loop E of the PS II intrinsic protein CP47 (loop E: residues ∼260–450) also resulted in a loss of photoautotrophic growth and reduction in PS II center assembly at pH 7.5 in some strains also lacking PsbV (**Table 1**). Alkaline pH 10.0 restored photoautotrophic growth (compared to pH 7.5) in ΔPsbV mutants carrying a CP47 Glu364 to Gln substitution or a deletion from Arg384 to Val392; however, ΔPsbV strains with either Phe363 of CP47 changed to Arg, or a deletion from Gly429 to Thr436, could not grow photoautotrophically at either pH level (Morgan et al., 1998; Clarke and Eaton-Rye, 1999; Eaton-Rye et al., 2003).

Mutations in loop E of CP47 are likely to contribute to a loss of growth in *Synechocystis* 6803 ΔPsbV strains by affecting assembly of the further extrinsic proteins to PS II. The C-terminal half of loop E of CP47 crosslinks with amino acids in the N-terminal region of PsbO and interacts with PsbU and possibly CyanoQ. Analysis of analogous amino acid residues from the *T. vulcanus* PS II crystal structure (Protein Data Base accession 4UB6) (Suga et al., 2015) using PyMOL^TM^ (Schrodinger, LLC; DeLano, 2002) show that the Arg384 to Val392 (Arg385-Val393 in *T. vulcanus*) region is within 4 Å of *T. vulcanus* PsbO Leu164-Gly167 and PsbU Asn11-Gly18; furthermore, a CP47 Δ(R384-V392):ΔPsbV mutant could not grow photoautotrophically at pH 7.5, possibly due to perturbation of PsbO and PsbU assembly to PS II. However, as noted above, the deletion of Gly429 to Thr436 in the ΔPsbV mutant resulted in a strain unable to grow at pH 7.5 or pH 10.0 – this more severe phenotype might result from impaired CyanoQ binding, in addition to perturbed PsbO binding. The Gly429-Thr436 (Gly427-Thr434 in *T. vulcanus*) residues are in close proximity (∼5.8 Å) to CP47 Asp440, and within 4 Å of *T. vulcanus* PsbO Gln176 and Lys178. CP47 Asp440 and PsbO Lys178 in *T. vulcanus* correspond to CP47 Asp440 and PsbO Lys180 in *Synechocystis* 6803, which were suggested to be important crosslinking sites for CyanoQ (Liu et al., 2014). Consistent with the hypothesis that impaired CyanoQ binding caused the loss of all photoautotrophic growth in the CP47 Δ(G429-T436):ΔPsbV strain, deletion of CyanoQ in the CP47 Δ(R384-V392):ΔPsbV background resulted in a strain that could not be rescued by pH 10.0. In the CP47 Δ(R384-V392) mutant, loss of PsbO did not cause pH sensitivity (**Table 1**): potentially these cells were already impaired in PsbO binding; therefore, deletion of PsbV in this strain might have resulted in a phenotype similar to the obligate photoheterotrophic ΔPsbO:ΔPsbV mutant (Shen et al., 1995).

## Proposed Effects of Environmental pH on PS II Mutants

### Cells Deficient in Extrinsic Proteins Are More Susceptible to Photoinhibition and Exhibit Reduced PS II Assembly at pH 7.5

The number of PS II centers was reduced by the loss of extrinsic proteins, particularly at pH 7.5 (**Table 1**). The extrinsic proteins stabilize the OEC and PS II dimer (Bricker et al., 2012), therefore, reduced PS II levels could be a result of altered PS II assembly processes. The external pH appears to have little impact on the lumenal pH in cyanobacteria (Belkin et al., 1987). However, a model describing connection between the thylakoid membrane and cytoplasmic membrane in cyanobacteria has been proposed to be via thylakoid centers that are involved in PS II biogenesis (Rast et al., 2015). Through their connection with the cytoplasmic membrane (Van de Meene et al., 2012) these thylakoid centers may be affected by the pH of the periplasm and this may alter PS II biogenesis.

Alternatively, or additionally, low levels of PS II centers in these mutants might be a consequence of the production of reactive oxygen species (ROS) by an impaired OEC; ROS cause photoinhibition by affecting PS II repair following photodamage, as well as by targeting PS II directly (Murata et al., 2007; Nixon et al., 2010; Vass, 2012). However, PS II centers lacking all, or specific combinations of extrinsic proteins are probably natural assembly and repair intermediates in the cyanobacterial cell. Assuming that PsbO and PsbV attach to PS II first, centers lacking PsbO and PsbU but retaining PsbV and CyanoQ (or lacking PsbV and CyanoQ, but retaining PsbO and PsbU) might not ordinarily occur, and could result in excess ROS leading to photoinhibition and a loss of photoautotrophic growth at low pH (**Figure 1A**). Some lines of evidence support this theory. Dissociation of the extrinsic proteins from spinach PS II-enriched membrane fragments resulted in increased hydrogen peroxide production (Hillier and Wydrzynski, 1993). In addition, a strain of *Synechococcus* sp. PCC 7942 lacking PsbU exhibited increased resistance to oxidative stress and this has been suggested to be due to increased ROS production associated with impaired PS II centers (Balint et al., 2006). Furthermore, although ΔPsbO:ΔPsbU cells, for example, cannot grow photoautotrophically at pH 7.5, cells supplemented with 5 mM glucose can grow and evolve oxygen from PS II when assayed with actinic light at 2.0 mE m^-2^ s^-1^ (Summerfield et al., 2005a). However, when 6.5 mE m^-2^ s^-1^ light is applied, rapid and total inactivation of oxygen evolution occurs in the same strains (Eaton-Rye et al., 2003). This implies that light dosage, as well as pH, causes the loss of PS II activity in these mutants. Additionally, cyanobacteria appear to be more sensitive to ROS at low pH; photomixotrophic growth of both the *Synechocystis* 6803 wild type and PS II mutants showed increased sensitivity to the ^1^O_2_ ROS generator Rose Bengal at pH 7.5 compared to pH 10.0 (Summerfield et al., 2013). ΔPsbO:ΔPsbU and ΔPsbV:ΔCyanoQ cells were also more sensitive to the O_2_^-^ generator methyl viologen than wild-type cells at either pH (Summerfield et al., 2013). At pH 10.0, cells may be able to better resist oxidative damage (**Figure 1B**); a suite of general oxidative stress-responsive genes were downregulated in ΔPsbO:ΔPsbU cells at pH 7.5 compared to pH 10.0, or compared to wild-type cells at either pH (Summerfield et al., 2013). Interestingly, a similar set of stress-responsive genes was upregulated in a ΔPsbO:ΔPsbU pseudorevertant capable of pH 7.5 growth (Summerfield et al., 2007), implying that antioxidant defense is involved in pH 7.5 recovery. Considering that total PS II levels are already reduced in these mutants, any photoinhibition by ROS at pH 7.5 might further reduce the number of effective PS II centers to levels that cannot sustain sufficient oxygen evolution and growth, especially in high light conditions. However, it must be considered that the relative level of PS II in these mutants does not correlate well with the capacity for photoautotrophic growth (**Table 1**) or oxygen evolution, indicating that more factors underpin the pH-sensitive phenotype than the capacity for PS II assembly alone.

### Reduced *Trans*-thylakoid pH Gradient at pH 7.5

During photosynthesis, the *trans*-thylakoid ΔpH is enhanced by proton release from light-driven oxidation of water by PS II. In PS II mutants, a reduction in assembled PS II centers, or a perturbation of water, oxygen and proton channels in the OEC would be likely to result in reduced proton delivery to the lumen. In cyanobacteria, ΔpH between the lumen and cytosol is as much as 2–3 pH units, and ΔpH increases with increasing environmental pH (Belkin et al., 1987). Therefore, reduced proton egress from PS II might be more harmful at pH 7.5 than pH 10.0, since ΔpH would already be reduced by environmental pH, potentially leading to ATP levels insufficient for cellular requirements (**Figure 1A**). While this theory is inconsistent with data on the pH-optimum for oxygen-evolving activity in extrinsic-protein deficient PS II centers isolated from higher plants (Commet et al., 2012), the function of isolated PS II in experimental conditions would be independent of cellular ATP and NADPH requirements. PS II mutants can grow in the presence of glucose at pH 7.5; respiration of added glucose could permit growth in these mutants by favoring CEF, allowing generation of ATP independently of PS II function. In *Synechococcus* Y-7c-s, a reduction in total cellular ATP, and the ATP:(ATP+ADP) ratio, was observed when external pH was reduced from pH 8 to growth-limiting pH 6 (Kallas and Castenholz, 1982). However, overall internal pH (the average of cytosol and thylakoid pH) across the same external pH range was only somewhat affected, suggesting that the observed limitation of energy supply might not be due to ΔpH alone. In support of the hypothesis that ATP supply might limit growth as pH is reduced, two strains of *Synechocystis* 6803 cells acclimated to pH 5.5 growth over 3 months independently acquired mutations in genes encoding F_1_–F_0_ ATP synthase components (Uchiyama et al., 2015), although these mutations are, as yet, functionally uncharacterized. Furthermore, experimental investigation of this hypothesis would require greater investigation of internal pH changes within the cell during photosynthesis and respiration, which to date has proven difficult (Berry et al., 2005).

As highlighted earlier, channels that surround the PS II OEC are likely to be perturbed by the loss of extrinsic proteins. Mutants with Phe363Arg and Glu364Gln substitutions in loop E of CP47 (**Table 1**) might also harbor impaired channels to the OEC. The *T. vulcanus* PS II crystal structure (*T. vulcanus* numbering is used throughout this paragraph) shows that CP47 Phe363 and Glu364 are adjacent to PsbO and D2 in a probable channel that might allow substrate water access to the OEC active site (Bricker et al., 2015). Additionally, Phe363 is implicated in the formation of a hydrophobic region around Y_D_ (D2 Tyr160), with the side-chain carboxyl group on Glu364 contributing to an H-bond network with Y_D_ via D2 Arg294 (Glu364-Arg294 distance: 2.8 Å; Ferreira et al., 2004; Saito et al., 2013; Suga et al., 2015). A perturbed hydrophobic pocket in the obligate photoheterotrophic F363R mutant might affect Y_D_ oxidation, altering the dark redox-state of Mn_4_CaO_5_ and resulting in a more deleterious phenotype than in the pH 7.5-sensitive E364Q mutant, where the carboxyl group on the substituted Gly might be able to partially contribute to H-bonding with D2 Arg294. Such speculation is only possible because of advances in the resolution of the PS II structure; experimental manipulation of putative channels in PS II in variable pH conditions has not been investigated as yet.

### Enhancement of Rubisco Activity by CO_2_ Draws Electrons from PS II and Reduces ROS Formation at pH 7.5

NADPH generated by photosynthetic electron transport is used to energize carbon uptake via Rubisco in the Calvin-Benson-Bassham (CBB) cycle; at pH 7.5, inorganic carbon is predominantly in the form of CO_2_ (for review, see Price, 2011; Kupriyanova et al., 2013). We observed that 3% CO_2_ supported photoautotrophic growth of ΔPsbO:ΔPsbU and ΔPsbV:ΔCyanoQ cells at pH 7.5, but had little impact on growth of the *Synechocystis* 6803 wild type, ΔPsbO:ΔPsbV cells (**Figures 1C,F**), or a ΔPsbO:ΔPsbU pseudorevertant (data not shown). In these conditions, increased Rubisco activity might require a large flux of NADPH, causing a build-up of NADP^+^; this major electron sink might accelerate photosynthetic electron transport, even where OEC function is impaired by the loss of extrinsic proteins in PS II mutants. Inhibitors of CBB cycle activity prevent repair of photodamaged PS II (Takahashi and Murata, 2005, 2008), therefore it is possible that enhanced CBB cycle activity at 3% CO_2_ has the opposite effect in ΔPsbO:ΔPsbU and ΔPsbV:ΔCyanoQ cells at pH 7.5, allowing efficient PS II repair and photoautotrophic growth (**Figure 1C**). In ambient CO_2_ conditions, without such a high demand for electrons from PS II, altered electron transfer in these mutants might otherwise result in production of ROS, and subsequently, photoinhibition (**Figure 1A**). At pH 10.0, total inorganic carbon and bicarbonate are somewhat higher compared to pH 7.5 (Price, 2011): hence the growth of some PS II mutants at pH 10.0 might also partially be a result of carbon fixation increasing the sink for electrons from PS II.

## Conclusion

A number of pH-sensitive *Synechocystis* 6803 PS II extrinsic protein mutants demonstrate that changes in environmental pH affect the function of the OEC and lumen-exposed PS II proteins. This is despite the presence of pH homeostasis mechanisms that buffer cytosolic pH and thylakoid lumen pH in cyanobacterial cells. Growth of these mutants at pH 10.0 appears to result in decreased ROS production, or increased oxidative stress responses; accordingly, light dosage appears to enhance the deleterious effects of pH 7.5 on oxygen evolution. Therefore, testing growth in pH 7.5 conditions is a useful investigative tool to reveal the relative stringency for different PS II proteins or protein regions; pH 10.0 conditions, in contrast, can be used to rescue some partially obligate photoheterotrophs carrying PS II mutations. The effects of environmental pH on PS II may arise through its impact on the requirement for substrate/product channels to and from the OEC that are dependent upon the extrinsic proteins. The environmental pH may also influence the extent of any destabilizing effect on PS II assembly that results from the absence or mutation of the PS II extrinsic proteins or lumenal regions of the transmembrane subunits. Increasing demand for NADPH, and hence photosynthetic electron transport, by elevated Rubisco activity might be a sink for excess energy captured by PS II with impaired function, thus allowing the recovery of some pH-sensitive strains by excess CO_2_. Understanding the causes of pH-sensitivity in such mutants may well elucidate the physiological mechanisms for maintaining an appropriate chemical environment for the shuttling of water, protons and oxygen to and from the Mn_4_CaO_5_ catalytic center.

## Author Contributions

JM wrote the first draft of the manuscript and all authors contributed to completing the manuscript.

## Conflict of Interest Statement

The authors declare that the research was conducted in the absence of any commercial or financial relationships that could be construed as a potential conflict of interest.
